# Sequential Enzymatic Hydrolysis and Ultrasound Pretreatment of Pork Liver for the Generation of Bioactive and Taste-Related Hydrolyzates

**DOI:** 10.1021/acs.jafc.4c02362

**Published:** 2024-07-02

**Authors:** Manuel Ignacio López-Martínez, Fidel Toldrá, Leticia Mora

**Affiliations:** Instituto de Agroquímica y Tecnología de Alimentos (CSIC), Avenue Agustín Escardino 7, 46980 Paterna, Valencia Spain

**Keywords:** pork liver, meat coproducts, sequential enzyme hydrolysis, ultrasound, taste, biological activities

## Abstract

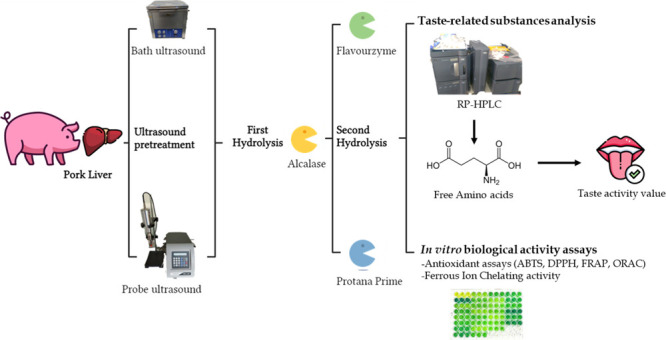

In the study of protein-rich byproducts, enzymatic hydrolysis stands as a prominent technique, generating bioactive peptides. Combining exo- and endopeptidases could enhance both biological and sensory properties. Ultrasound pretreatment is one of the most promising techniques for the optimization of enzymatic hydrolysis. This research aimed to create tasteful and biologically active pork liver hydrolyzates by using sequential hydrolysis with two types of enzymes and two types of ultrasound pretreatments. Sequential hydrolyzates exhibited a higher degree of hydrolysis than single ones. Protana Prime hydrolyzates yielded the largest amount of taste-related amino acids, enhancing sweet, bittersweet, and umami amino acids according to the Taste Activity Value (TAV). These hydrolyzates also displayed significantly higher antioxidant activity. Among sequential hydrolyzates, Flavourzyme and Protana Prime hydrolyzates pretreated with ultrasound showed the highest ferrous ion chelating activity. Overall, employing both Alcalase and Protana Prime on porcine livers pretreated with ultrasound proved to be highly effective in obtaining potentially tasteful and biologically active hydrolyzates.

## 1 Introduction

One of the main current challenges in the food industry is the increasing generation of byproducts, which frequently leads to serious economic and environmental impacts.^1^ However, some food byproducts are rich in bioactive substances with high added value. In this respect, fruit and vegetable byproducts are rich in fiber and vitamins,^1^ while meat byproducts and coproducts are rich in vitamins, minerals, and proteins of high biological value.^2,3^ Enzymatic hydrolysis is one of the most widely used techniques for the obtention of bioactive peptides from food byproduct proteins.^1^

Bioactive peptides are sequences of 2 to 20 amino acids, which have no biological function in the native protein but may exert a beneficial effect on health when they are released from the native protein.^4^ It has been widely studied that bioactive peptides may exert biological activities such as antioxidant, antihypertensive, or immunomodulatory effects.^4^ In addition, it has been shown that certain raw materials such as meat coproducts like liver, di- and tripeptides, and free amino acids can be mainly responsible for taste.^3,5^ Therefore, enzymatic hydrolysis could favor the development of functional ingredients with enhanced biological activity and sensory properties, which would make them ideal for inclusion in a functional food.

Commercial proteases are obtained from many sources like fruits (papain and bromelain), animal gastric organs (pepsin or trypsin), or microbes (Alcalase from *Bacillus licheniformis* or Flavourzyme from *Aspergillus oryzae*) and can be divided into exo- and endopeptidases.^2^ Endopeptidases act inside the protein matrix enhancing the release of large protein fragments, polypeptides, and peptides, while exopeptidases exert their effect in the N and C terminals of proteins and peptides, promoting the release of small peptides and free amino acids.^6^ In general, the combination of endo- and exopeptidases favors the release of bioactive peptides and free amino acids.^7^

Despite the benefits of using the enzymatic hydrolysis of proteins for the generation of bioactive peptides, the industrial production of these proteases is currently very expensive. For this reason, the use of pretreatment technologies that could enhance the enzymatic hydrolysis of proteins, such as microwaves, high hydrostatic pressure, or ultrasound is currently being investigated.^7^ It has been observed that the use of ultrasounds can generate the unfolding in the protein structure, releasing more access points for the enzyme to hydrolyze, leading to an optimization of the process by reducing hydrolysis times and the amount of enzyme used.^8^

The main objective of this research was to study the effect of using sequential enzymatic hydrolysis and ultrasound pretreatments on the generation of taste-related and bioactive hydrolyzates from pork livers.

## 2 Materials and Methods

### 2.1 Chemicals and Reagents

All high-performance liquid chromatography (HPLC) reagents (acetonitrile (ACN), methanol (MeOH), and ethanol (EtOH)) were purchased from Scharlau (Barcelona, Spain). All enzymes (Alcalase 4.0L Pure, Flavourzyme 1000L, and Protana Prime) were acquired from Novozymes (Bagsværd, Denmark).

Sodium acetate, monobasic sodium phosphate, disodium tetraborate decahydrate, potassium dihydrogen orthophosphate, ethylenediaminetetraacetic acid (EDTA), and ascorbic acid were obtained from PanReac (Darmstadt, Germany). Potassium ferricyanide, potassium persulfate, ferric chloride, trichloroacetic acid (TCA), butylated hydroxytoluene (BHT), 2,2-diphenyl-1-picryl-hydrazyl-hydrate (DPPH), 2,2′-azino-bis 3-ethylbenzothiazoline-6-sulfonic acid (ABTS), o-phthalaldehyde, 2-beta mercaptoethanol, sodium dodecyl sulfate (SDS), iron(II) chloride tetrahydrate, sodium 4-[3-(pyridin-2-yl)-6-(4-sulfophenyl)-1,2,4-triazin-5-yl]benzene-1-sulfonate (Ferrozine), 6-hydroxy-2,5,7,8-tetramethylchroman-2-carboxylic acid (Trolox), fluorescein, 2,2′-azobis(2-methylpropionamidine) dihydrochloride (AAPH), DL-Ditiotreitol (DTT) and amino acid standards were purchased from Sigma (St.Louis, MO, USA). A precast electrophoresis gel kit was acquired from Bio-Rad (California, USA).

### 2.2 Sample Preparation and Enzymatic Hydrolysis Procedure

#### 2.2.1 Sample Preparation

Pork livers were purchased from a porcine slaughterhouse. To avoid the action of endogenous enzymes, livers were cut into pieces and subjected to heat treatment in a thermostatic bath at 85 °C for 10 min. Samples were minced (Moulinette with reference A320R1, Moulinex, France), and the resulting paste was diluted 1:1 (m/v) with bidistilled water. The mixture was homogenized in a masticator homogenizer (IUL Mod. Masticator Classic Panoramic, 400 mL, Microplanet, Spain) for 5 min at 4 °C. Finally, the pH was adjusted to 8 with 10 M NaOH.

#### 2.2.2 Proximate Composition and Physicochemical Properties

pH was measured with a meat pHmeter (HI 99163, HANNA instruments, USA) and water activity (*a*_w_) with an *a*_w_ meter (Aqualab 4Tev, Meter Food, USA) at 25 °C. Moisture of livers was obtained using a thermobalance (HB43 Halogen, Metter Toledo, USA), and protein content was determined by the Dumas combustion method according to AOAC method 992.15.^9^

#### 2.2.3 Ultrasound Pretreatment

An ultrasound pretreatment was performed in 250 mL of 1:1 pork liver/bidistilled water dilution before the enzymatic hydrolysis. Two ultrasound devices were tried: (i) an ultrasonic probe (Sonics Materials VCX-750–220, Thermofisher, USA) under the conditions of frequency 20 kHz, amplitude 75%, 90 min of time, and pulses 2 s on/4 s off and (ii) an ultrasonic bath (Transsonic TI-H, VWR, USA) under the parameters of frequency 35 kHz, amplitude 100%, and 120 min of time without interval pulses.

#### 2.2.4 First Enzymatic Hydrolysis

The first hydrolysis procedure was performed under the following conditions recommended by the manufacturer: 2% Alcalase 4.0 L pure for 2 h at 65 °C under constant agitation (500 rpm).

#### 2.2.5 Second Enzymatic Hydrolysis

##### 2.2.5.1 Flavourzyme Second Hydrolysis

The Flavourzyme second hydrolysis procedure was carried out following the conditions recommended by the manufacturer: 5% Flavourzyme 1000 L for 2 h at 50 °C under constant stirring of 500 rpm.

##### 2.2.5.2 Protana Prime Second Hydrolysis

The Protana Prime second hydrolysis procedure was executed according to the conditions recommended by the manufacturer: 5% Protana Prime for 16 h (overnight), at 55 °C under constant agitation of 500 rpm.

After the second hydrolysis, the hydrolyzates were inactivated at 85 °C for 10 min in a thermostatic bath. Then, samples were cooled in an ice bath and stored at −20 °C until use. All tested conditions and their codifications are shown in Table 1.

**Table 1:** Codification of the Conditions of Pork Liver Hydrolyzates

samples	ultrasound pretreatment	1st enzyme	2nd enzyme	hydrolysis time (min)
B	none	none	none	0
BUB	bath			
BUP	probe			
SH	none	Alcalase 4.0 L	none	120
SHUB	bath			
SHUP	probe			
DHF	none	Alcalase 4.0 L	Flavourzyme 1000 L	240
DHFUB	bath			
DHFUP	probe			
DHP	none	Alcalase 4.0 L	Protana Prime	overnight
DHPUB	bath			
DHPUP	probe			

### 2.3 Degree of Hydrolysis (DH)

The degree of hydrolysis (DH) was analyzed following the Nielsen et al. method.^10^ In short, 36 μL of the sample was mixed with 270 μL of OPA reagent (200 mL of bidistilled water, 7.620 g of disodium tetraborate decahydrate solution, 0.2 g of SDS, 4 mL of 40 mg/mL o-phthalaldehyde solution in methanol, and 0.176 g of DTT). The mixture was incubated for 2 min at 25 °C. The absorbance was determined at a wavelength of 340 nm using a plate reader (CLARIOstar Plus, BMG Labtech, Germany). The degree of hydrolysis was calculated as follows:
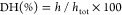
where *h*_tot_ is the total peptide linkages per protein equivalent and is determined by the amino acid composition of the protein of origin and *h* represents the number of these bonds that were hydrolyzed. In this case, the parameters of meat proteins were used to calculate the degree of hydrolysis.

All assays were performed in triplicate, and mean values were reported.

### 2.4 Determination of Free Amino Acids (FAAs)

A dilution of 1/100 (v:v) with HCl 0.01 N was carried out for sample preparation. The derivatization was done following the Aristoy & Toldra, methodology^11^ using 5 mM norleucine solution as the internal standard. A reverse-phase HPLC system (Acquity Arc CH/CHC Core Fluidics; Waters, USA) equipped with a Waters Pico Tag C18 column (60 Å, 4 μm, 3.9 mm × 300 mm; Waters Corp., Milford, MA, USA) was used for the chromatographic analysis following the Flores et al., method.^12^ 70 mM sodium acetate with 2.5% ACN at pH 6.55 and ACN/H_2_O/MeOH in a ratio of 45:40:15 were used as mobile phases. The conditions of the analysis were as follows: sample temperature of 12 °C, column temperature of 52 °C, flow rate of 1 mL/min, and detection wavelength monitored at 254 nm. Quantification was carried out using standard curves for each amino acid. The results were expressed as mg of FAAs/g of the sample. Taste attributes of amino acids were compiled using data published elsewhere.^13−15^

### 2.5 Taste Activity Value (TAV)

The taste activity value (TAV) is a parameter that indicates the influence of a single substance on the overall taste of a food and it is defined by the ratio between the concentration of the compound in the product (*C*) and its taste threshold (*T*)^13^:
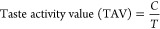
TAV values higher than 1 mean that the analyte contributes actively to the taste, and the higher this value, the greater its effect on food′s taste. Taste thresholds were taken from published data.^13,15,16^ The TAVs are dimensionless and were made in triplicate for each sample, and the average values were reported.

### 2.6 SDS-PAGE Electrophoresis Assay

For sample preparation, an appropriate volume of Laemmli sample buffer with 2-beta-mercaptoethanol was added to 8 μL of 1:10 dilutions of samples with bidistilled water.

The samples were submitted to thermal treatment (95 °C, 5 min) to achieve their denaturation and then added to electrophoresis gel wells. Electrophoresis was carried out using an AnyKD Bio-Rad gel at 200 V for 30 min. The gel was fixed with 40% ethanol/10% acetic acid for 1 h, and then colloidal Coomassie (Bio-Rad) was used to stain the gel for 1 h more. The gel was then destained with distilled water, and the gel image was scanned with an Image Scanner (GE).

### 2.7 Biological Activity Assays

#### 2.7.1 Sample Preparation

Three volumes of EtOH were added to 100 μL of all samples to carry out deproteinization during 16 h.^17^ Samples were then centrifuged at 4 °C for 5 min at 20,879 *g*. The supernatants were collected and used to carry out biological activity experiments.

#### 2.7.2 Antioxidant Activity Assays

##### 2.7.2.1 DPPH Free Radical-Scavenging Activity

The DPPH free radical-scavenging activity was measured following the method of Bersuder et al.^18^ In brief, a mixture of 100 μL of sample or control, 500 μL of ethanol, and 125 μL of DPPH solution (0.2 mM in ethanol) was made. Ethanol was the negative control, and BHT at 0.02 mg/mL in ethanol was the positive control. The samples were incubated in the dark at 25 °C for 60 min, and absorbance was obtained at 517 nm using a UV–vis spectrophotometer (Cary 60 UV–visible spectrophotometer, Agilent Technologies, CA, USA).

The DPPH radical-scavenging activity was calculated using the following equation:
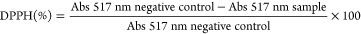


All assays were executed in triplicate, and results were expressed as mean values.

##### 2.7.2.2 ABTS Radical-Scavenging Capacity

The ABTS assay was carried out following the Re et al., method^19^ with some modifications. A mixture of 10 μL of sample and 990 μL of ABTS working solution was made and incubated for 6 min in the dark at 25 °C. The absorbance of the samples was measured at 734 nm using a UV–visible spectrophotometer (Cary 60 UV–visible spectrophotometer, Agilent Technologies, CA, USA). Ascorbic acid was the positive control, and PBS 50 mM at pH 7.4 was the negative. Besides, different concentrations of Trolox (0.05–2.5 mM) were used as standard curves. The measurements were made in triplicate, and the average values were reported as mmol of TEAC (Trolox Equivalent Antioxidant Capacity) per mg of sample.

##### 2.7.2.3 Ferric Reducing Antioxidant Power (FRAP) Assay

The FRAP assay was analyzed following the Chen et al., method^20^ with slight modifications. In brief, 140 μL of each sample or control was mixed with 140 μL of 0.2 M sodium phosphate buffer (PBS) (pH 6.6) and 140 μL of 1% (w/v) potassium ferricyanide and incubated for 20 min at 50 °C. 140 μL of 10% (w/v) TCA was then added to the mixture, and then, the samples were centrifuged for 10 min at 200 × *g*; 400 μL of supernatant were collected and mixed with 80 μL of 1% (w/v) ferric chloride and 400 μL of distilled water. The reaction was performed in the dark for 10 min. After incubation, the absorbance of samples was obtained at 700 nm using a UV–visible spectrophotometer (Cary 60 UV–visible spectrophotometer, Agilent Technologies, CA, USA). PBS 0.2 M at pH 6.6 was used as a negative control, and BHT at 0.02 mg/mL in ethanol was used as a positive control. Higher values of absorbance are correlated with higher reducing power. The assay was carried out in triplicate, and mean values were reported as absorbance units.

##### 2.7.2.4 Oxygen Radical Absorbance Capacity (ORAC) Assay

The oxygen radical absorbance capacity (ORAC) assay was performed according to the Davalos et al., methodology.^21^ In short, 140 μL of each liver sample and 70 μL of a 200 nM fluorescein solution diluted in PBS at pH 7.4 were added into a black-bottom 96-well plate. After 15 min of incubation in the dark at 37 °C, 70 μL of 80 mM AAPH diluted in PBS pH 7.4 was added. Fluorescence emission was continuously monitored over a duration of 95 min within a controlled thermal environment of 37 °C using a plate reader (CLARIOstar Plus, BMG Labtech, Germany). The excitation wavelength was set at 485 nm, while the emission wavelength was fixed at 520 nm. Normalized fluorescence curves were obtained, and subsequently, the area under the fluorescence decay curve (AUC) was computed for individual wells by utilizing the following mathematical expression:
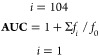


The formula for computing the area under the fluorescence decay curve (AUC) involves the use of initial fluorescence reading (*f*_0_) at time 0 min and subsequent readings (*f*_i_) at various time intervals (*i* minutes). Standard curves were generated using different concentrations of Trolox (16 to 0.2 μM), while PBS 50 mM at pH 7.4 was employed as a negative control and tryptophan 1.5 μM solution in PBS 7.4 as a positive control. Results were expressed as mmol of Trolox equivalent/g of liver. All assays were carried out in triplicate, and results were reported as mean values.

#### 2.7.3 Ferrous Ion Chelating Activity

The ferrous ion chelating activity was measured according to the Zheng et al., method^22^ with slight modifications; 50 μL of the sample or control was introduced into a transparent-bottom 96-well plate with 25 μL of 0.2 mM FeCl_2_ solution and 100 μL of bidistilled water. The positive control was EDTA 1 mg/mL solution, and the negative control and blank (without reagents) were bidistilled water. These mixtures were incubated in the dark for 3 min; 100 μL of 0.5 mM ferrozine solution was added, and then, the samples were incubated in the dark for 10 min. The absorbance of the samples was measured at 562 nm in a plate reader (CLARIOstar Plus, BMG Labtech, Germany).

The ferrous ion chelating activity in % was obtained following the next equation:
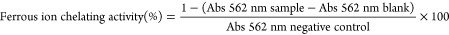


All assays were performed in triplicate, and results are reported as mean values.

### 2.8 Statistical Analysis

The statistical analysis of the data was performed by an analysis of variance (ANOVA) followed by Tukey’s study range test, with a significance level of *p* < 0.05. Minitab (Minitab, LLC, USA) was the statistic program used for this purpose.

Capital letters mean significant differences among the results of the same group for each parameter, while lower-case letters mean significant differences among all the results for each parameter.

## 3 Results

### 3.1 Proximate Composition

Fresh porcine livers showed a pH of 6.68 ± 0.05, *a*_w_ of 0.994 ± 0.001, with 73.05 ± 1.68% of moisture, and 20.96 ± 0.44% of protein. These results are consistent with those obtained by other authors.^3,23^

### 3.2 Degree of Hydrolysis and SDS-Page Electrophoresis

**Figure 1:**
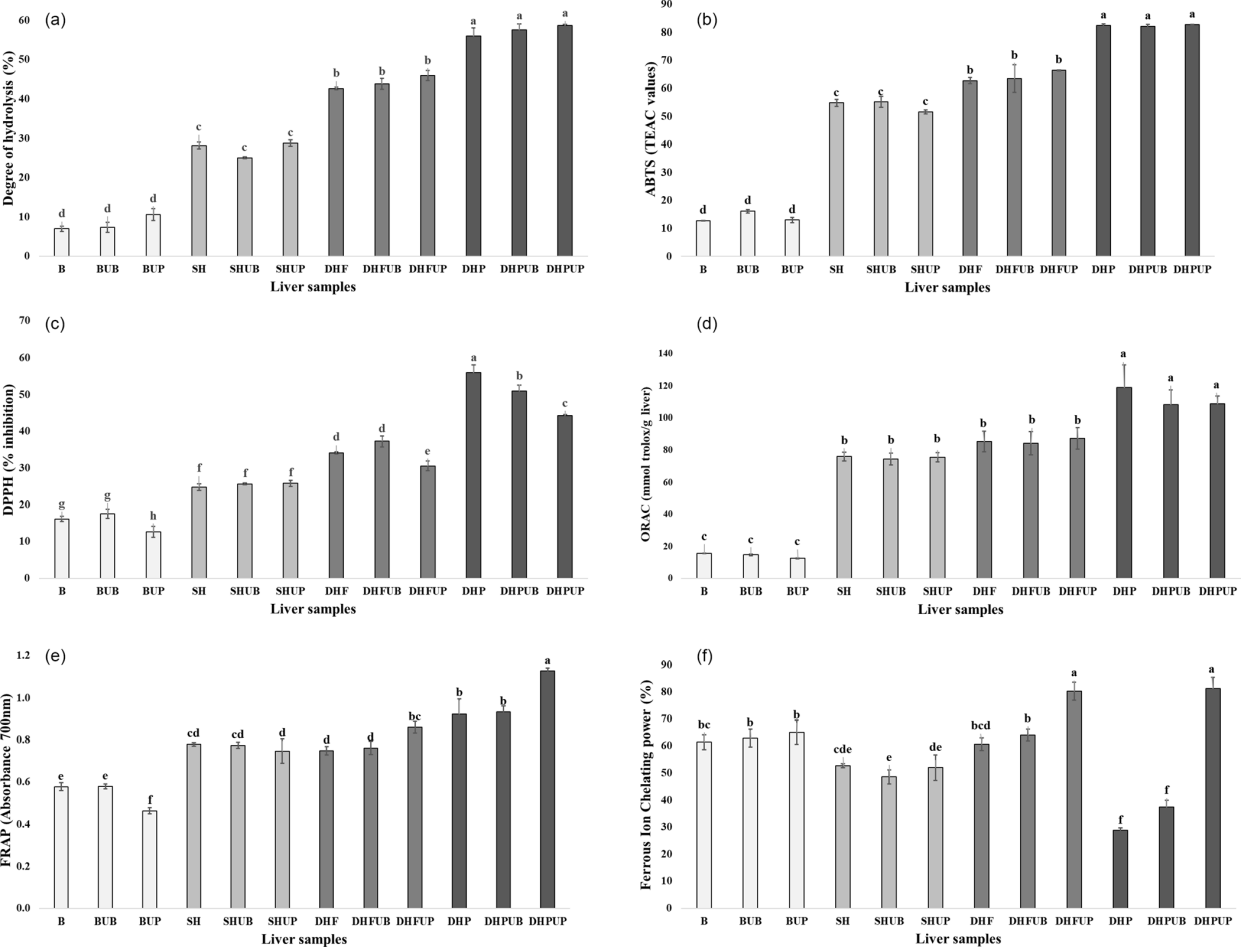
Biological activities and degree of hydrolysis of liver samples. (A) Degree of hydrolysis in % by the OPA method, (B) ABTS assay expressed in TEAC values, (C) DPPH assay expressed in % of DPPH inhibition, (D) ORAC assay expressed in mmol Trolox/g liver, (E) FRAP assay expressed in absorbance values at 700 nm, and (F) ferrous chelating activity power expressed as % of chelation. Results are expressed by mean ± SEM of triplicates.

**Figure 2:**
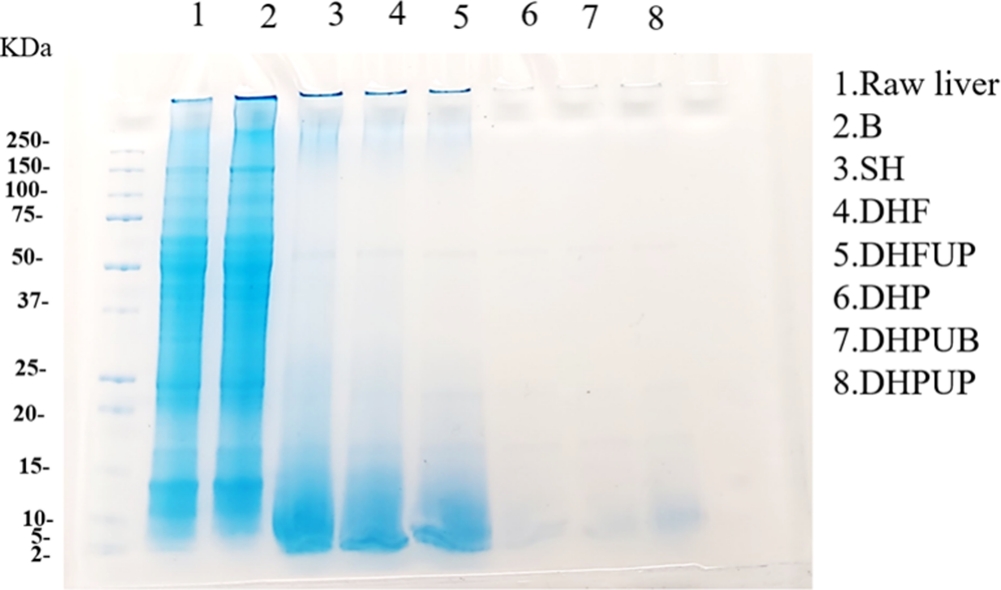
SDS-Page electrophoresis gel of liver samples.

According to Figure 1A, Protana Prime showed the highest DH, followed by Flavourzyme sequential hydrolyzates, single hydrolyzates, and unhydrolyzed samples. These results are generally consistent with the published literature, as the samples subjected to sequential hydrolysis treatment have significantly higher values than those that were hydrolyzed using only one enzyme (*p* < 0.05).^24,25^Figure 2 shows a decrease in the intensity of color bands in single hydrolysis (SH) compared to the control (raw liver and B), mainly due to the intense hydrolysis and production of peptides lower than 20 kDa. Flavourzyme hydrolysis also reduced the intensity of bands between 75 and 20 kDa, which could be related to the increase in DH. Protana Prime hydrolyzates showed a very intense hydrolysis compared to the other hydrolyzates, although DHPUP showed some bands within the range of 10 to 2 kDa in comparison with DHPUB and DHP hydrolyzates.

Some studies have shown that ultrasound improves the degree of hydrolysis of the hydrolyzates because it promotes structural changes in the protein through cavitation, favoring the appearance of cutoff sites for the enzyme.^8,26^ Meanwhile, it has also been pointed out that prolonged ultrasonic treatment may induce overheating of the sample which, in addition to the hydrolysis inactivation heat treatment, could favor the aggregation of the protein structure, reducing the possibility for the protease action.^27,28^ In this study, the possible unfolding or aggregation of the native proteins generated by ultrasound is not perceived since this pretreatment does not increase the degree of hydrolysis among all groups of samples. Therefore, it seemed that the effect of the enzyme overshadowed the effect of the ultrasound.

### 3.3 Free Amino Acid Content and Taste Activity Value

**Figure 3:**
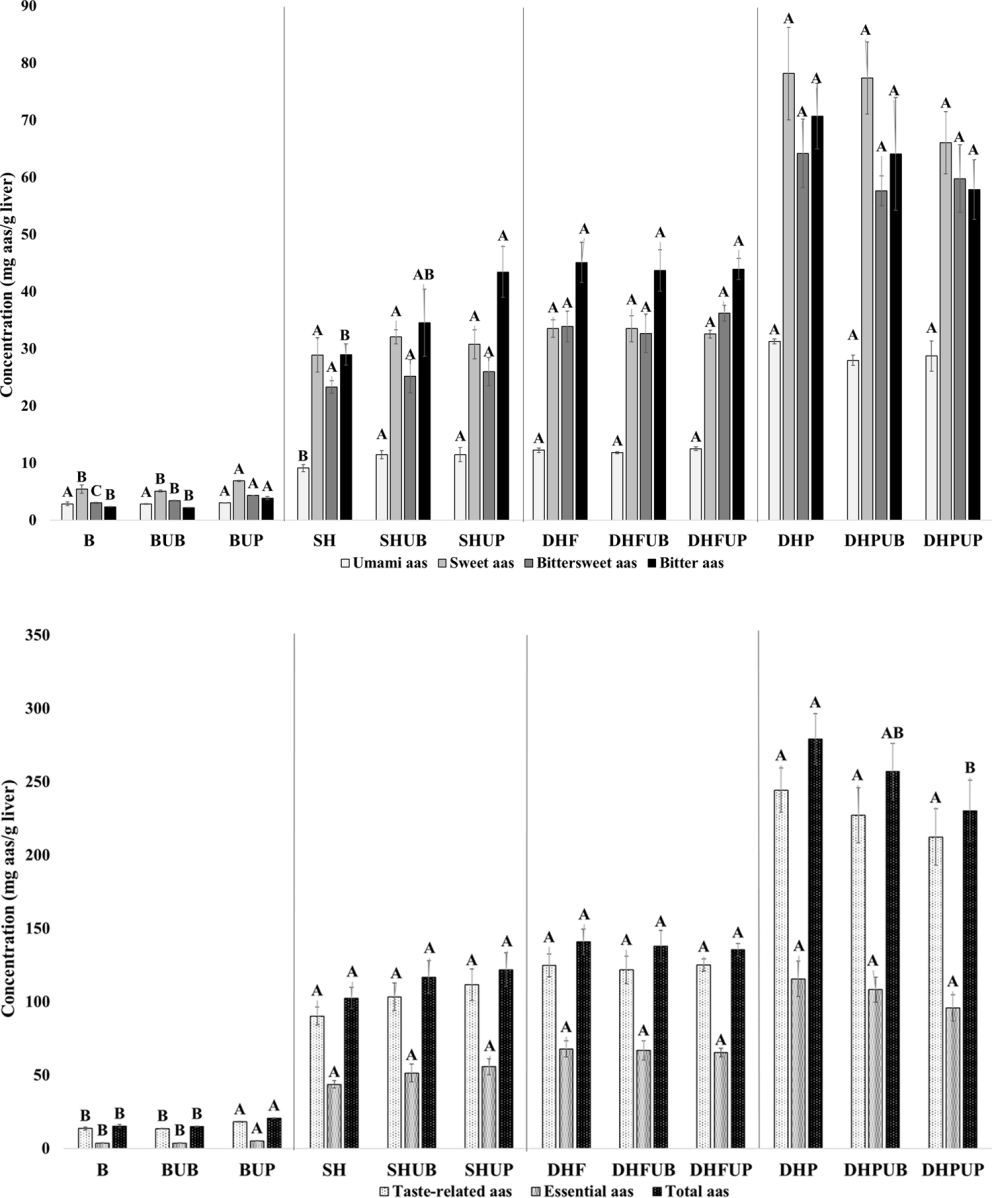
Summatory of free amino acids (mg amino acid/g liver) in liver samples separated by groups (unhydrolyzed, Alcalase hydrolyzates, Flavorzyme sequential hydrolyzates, and Protana Prime sequential hydrolyzates). (A) Summatory of taste attributes (umami, sweet, bittersweet, and bitter) and (b) total content of taste-related, essential, and total amino acids. Results are expressed as mean ± SEM of triplicates, and statistics were only performed within the groups and not comparing among them.

Table 2 shows the free amino acid content of the liver samples. Regarding umami amino acids, Protana Prime sequential hydrolyzates showed the highest concentration, releasing up to 4 times more Asp (2.885 to 12.449 mg aas/g liver) and 3 times more Glu (6.257 to 18.856 mg aas/g liver) and total umami amino acids (9.142 to 31.305 mg aas/g liver) than single hydrolyzates. The effect of ultrasound was not significant among all samples, but when it was compared within the same group (Figure 3A), a significant increase in total umami amino acid content was observed in SHUP and SHUB with respect to SH (*p* < 0.05), probably because the US pretreatment only enhanced umami in single hydrolyzates. In the case of sweet amino acids, a significant increment was perceived for Ser, Gly, Ala, Thr, and total sweet amino acids in all Protana Prime hydrolyzates in comparison to single hydrolyzates (*p* < 0.05). Observing bittersweet amino acids, a significant increase in total bittersweet amino acid content was reported in all Protana Prime hydrolyzates and in DHF and DHFUP compared with single hydrolyzates (*p* < 0.05). In addition, Met content in all Flavourzyme hydrolyzates is 2 times higher than in Protana Prime hydrolyzates (10.884 > 5.309 mg aas/g liver).

**Table 2:** Free Amino Acids of Liver Hydrolyzates Expressed as mg/g Liver (N=3, Mean±SEM).

liver samples	B	BUB	BUP	SH	SHUB	SHUP	DHF	DHFUB	DHFUP	DHP	DHPUB	DHPUP
mg aas/g liver	mean ± SEM	mean ± SEM	mean ± SEM	mean ± SEM	mean ± SEM	mean ± SEM	mean ± SEM	mean ± SEM	mean ± SEM	mean ± SEM	mean ± SEM	mean ± SEM
Asp (Um)^(1,2,3)^^a^	0.913 ± 0.169c	0.902 ± 0.032c	0.924 ± 0.056c	2.885 ± 0.269bc	3.449 ± 0.424b	2.838 ± 0.311bc	3.703 ± 0.456b	3.617 ± 0.491b	3.460 ± 0.044b	12.449 ± 2.082a	12.089 ± 0.856a	11.881 ± 1.079a
Glu (Um)^(1,2,3)^	1.964 ± 0.194d	1.942 ± 0.021d	2.135 ± 0.048d	6.257 ± 0.618c	8.052 ± 0.956c	8.667 ± 0.940c	8.574 ± 0.497c	8.213 ± 0.694c	9.068 ± 0.289c	18.856 ± 2.493a	15.918 ± 0.179b	16.868 ± 1.584ab
**umami aas**	2.877 ± 0.346**e**	2.844 ± 0.051**e**	3.059 ± 0.008**e**	9.142 ± 0.638**d**	11.501 ± 0.708**cd**	11.505 ± 1.249**cd**	12.278 ± 0.389**c**	11.830 ± 0.205**cd**	12.528 ± 0.333**c**	31.305 ± 0.427**a**	28.007 ± 0.879**b**	28.749 ± 2.659**ab**
Ser (Sw)^(1,2,3)^	0.894 ± 0.135d	0.854 ± 0.041d	1.256 ± 0.021d	5.638 ± 0.612c	6.213 ± 0.749c	5.962 ± 0.320c	6.854 ± 0.439c	6.780 ± 0.365c	6.752 ± 0.298c	13.423 ± 1.691a	11.185 ± 0.586b	13.066 ± 1.227ab
Gly (Sw)^(1,2,3)^	2.393 ± 0.364bc	2.144 ± 0.234c	2.246 ± 0.053c	5.366 ± 0.533bc	5.750 ± 0.543b	3.585 ± 0.158bc	4.412 ± 0.398bc	4.304 ± 0.331bc	4.220 ± 0.179bc	18.188 ± 3.571a	16.233 ± 0.334a	18.215 ± 1.328a
Gln (Sw)^(3)^	0.336 ± 0.027d	0.261 ± 0.006d	0.738 ± 0.023d	3.496 ± 0.480c	4.259 ± 0.743bc	6.271 ± 0.596a	5.837 ± 0.685a	5.742 ± 0.496a	6.091 ± 0.212a	5.262 ± 0.552ab	5.415 ± 0.092ab	3.677 ± 0.409c
Thr (Sw)^(1,2,3)^	0.409 ± 0.015e	0.431 ± 0.029e	0.798 ± 0.023e	4.864 ± 0.406d	5.720 ± 0.667d	5.857 ± 0.560d	6.485 ± 0.742d	6.700 ± 0.703d	6.374 ± 0.246d	14.615 ± 3.067b	18.879 ± 1.483a	10.488 ± 0.698c
Ala (Sw)^(1,2,3)^	1.438 ± 0.247c	1.449 ± 0.173c	1.847 ± 0.012c	9.588 ± 1.294b	10.179 ± 1.306b	9.126 ± 0.917b	9.980 ± 0.508b	10.010 ± 0.606b	9.150 ± 0.298b	26.690 ± 4.712a	25.711 ± 5.547a	20.629 ± 1.819a
**sweet aas**	5.470 ± 0.707**d**	5.139 ± 0.179**d**	6.885 ± 0.120**d**	28.953 ± 3.026**c**	32.120 ± 1.245**c**	30.801 ± 2.534**c**	33.568 ± 1.539**c**	33.535 ± 2.273**c**	32.587 ± 0.671**c**	78.179 ± 8.091**a**	77.422 ± 6.343**a**	66.075 ± 5.451**b**
Arg (BS)^(2,3)^	0.042 ± 0.009f	0.550 ± 0.053def	0.808 ± 0.058d	0.392 ± 0.065def	0.235 ± 0.014ef	2.060 ± 0.401c	0.839 ± 0.046d	0.649 ± 0.066de	5.415 ± 0.239b	0.049 ± 0.002f	0.240 ± 0.026ef	6.171 ± 0.388a
Pro (BS)^(2,3)^	0.681 ± 0.080d	0.664 ± 0.041d	0.965 ± 0.032 cd	0.714 ± 0.039d	0.776 ± 0.059 cd	1.092 ± 0.031 cd	1.310 ± 0.011 cd	1.307 ± 0.040 cd	1.099 ± 0.032 cd	3.699 ± 0.662a	2.735 ± 0.394b	1.445 ± 0.110c
Val (BS)^(2,3)^	0.664 ± 0.004d	0.641 ± 0.044d	0.565 ± 0.060d	8.464 ± 0.344c	8.480 ± 1.150c	9.522 ± 0.969c	7.358 ± 0.570c	6.788 ± 0.484c	6.753 ± 0.653c	21.247 ± 2.483a	20.829 ± 3.583ab	16.846 ± 1.937b
Lys (BS)^(2,3)^	1.385 ± 0.078c	1.327 ± 0.043c	1.619 ± 0.020c	10.753 ± 1.176b	12.277 ± 1.158b	9.716 ± 0.974b	13.570 ± 1.310b	13.172 ± 1.533b	12.429 ± 0.802b	33.932 ± 7.154a	29.462 ± 0.518a	30.734 ± 3.109a
Met (BS)^(2,3)^	0.270 ± 0.035d	0.262 ± 0.019d	0.420 ± 0.012d	2.989 ± 0.325c	3.448 ± 0.722bc	3.614 ± 0.318bc	10.844 ± 0.868a	10.807 ± 1.281a	10.558 ± 0.355a	5.309 ± 0.939b	4.385 ± 0.828bc	4.609 ± 0.429bc
**bittersweet aas**	3.040 ± 0.083**e**	3445 ± 0.097**e**	4.377 ± 0.009**e**	23.313 ± 1.106**d**	25.215 ± 2.881**cd**	26.004 ± 2.456**cd**	33.921 ± 2.708**bc**	32.722 ± 3.361**bc**	36.254 ± 1.394**b**	64.234 ± 6.018**a**	57.651 ± 2.619**a**	59.804 ± 5.920**a**
Tau (Bt)^(1,3)^	0.352 ± 0.019ef	0.219 ± 0.042fg	1.032 ± 0.095a	0.144 ± 0.000g	0.157 ± 0.019g	1.069 ± 0.100a	0.663 ± 0.015c	0.643 ± 0.018c	1.006 ± 0.041a	0.534 ± 0.013 cd	0.395 ± 0.084de	0.845 ± 0.041b
His (Bt)^(1,2,3)^	0.064 ± 0.010d	0.060 ± 0.009d	0.744 ± 0.009c	1.489 ± 0.054b	1.745 ± 0.141b	3.536 ± 0.471a	3.370 ± 0.062a	3.354 ± 0.178a	3.582 ± 0.116a	3.639 ± 0.248a	3.113 ± 0.240a	3.491 ± 0.267a
Tyr (Bt) ^(1,2,3)^	0.256 ± 0.035e	0.234 ± 0.016e	0.405 ± 0.082e	3.712 ± 0.401d	4.349 ± 0.935 cd	5.793 ± 0.657bc	7.358 ± 0.570ab	6.788 ± 0.484ab	6.753 ± 0.653ab	8.241 ± 0.842a	8.133 ± 1.609a	6.855 ± 0.422ab
Ile (Bt)^(1,2,3)^	0.422 ± 0.017e	0.441 ± 0.014e	0.396 ± 0.016e	5.697 ± 0.629 cd	6.351 ± 1.108 cd	6.819 ± 0.661c	4.600 ± 0.368 cd	4.444 ± 0.418 cd	3.943 ± 0.211d	14.760 ± 1.580a	13.099 ± 2.190ab	11.700 ± 1.267b
Leu (Bt)^(1,2,3)^	0.845 ± 0.023d	0.851 ± 0.063d	0.859 ± 0.050d	11.045 ± 1.209bc	14.113 ± 2.859b	15.529 ± 1.502b	8.119 ± 0.650c	7.897 ± 0.729c	7.855 ± 0.358c	28.277 ± 3.188a	25.536 ± 4.531a	22.539 ± 2.069a
Phe (Bt)^(1,2,3)^	0.356 ± 0.025e	0.372 ± 0.037e	0.317 ± 0.025e	5.313 ± 0.640d	5.969 ± 1.048d	7.946 ± 0.786 cd	18.004 ± 1.456a	17.471 ± 1.710a	17.802 ± 1.246a	11.798 ± 1.094b	10.707 ± 1.194bc	9.488 ± 0.926bc
Trp (Bt)^(1,2,3)^	0.069 ± 0.016c	0.042 ± 0.007c	0.134 ± 0.011c	1.605 ± 0.351b	1.914 ± 0.140b	2.778 ± 0.286a	3.030 ± 0.420a	3.130 ± 0.182a	3.028 ± 0.186a	3.463 ± 0.496a	3.176 ± 0.207a	2.959 ± 0.249a
**bitter aas**	2.363 ± 0.023**e**	2.218 ± 0.045**e**	3.886 ± 0.241**e**	29.005 ± 1.849**d**	34.597 ± 5.899**cd**	43.470 ± 4.459**c**	45.144 ± 3.513**bc**	43.727 ± 3.615**c**	43.969 ± 1.848**c**	70.711 ± 5.732**a**	64.158 ± 9.877**a**	57.877 ± 5.228**ab**
Hpro	0.046 ± 0.002c	0.045 ± 0.004c	0.300 ± 0.010ab	0.015 ± 0.005c	0.126 ± 0.150c	0.309 ± 0.029a	0.312 ± 0.017a	0.300 ± 0.021ab	0.315 ± 0.010a	0.276 ± 0.065ab	0.155 ± 0.029bc	0.031 ± 0.004c
Asn	0.276 ± 0.002f	0.306 ± 0.007f	0.783 ± 0.051f	3.589 ± 0.365e	4.306 ± 0.941de	5.810 ± 0.399 cd	6.688 ± 0.086c	6.314 ± 0.444c	6.406 ± 0.190c	10.446 ± 1.170a	8.563 ± 0.111b	9.376 ± 0.759ab
β-Ala	0.205 ± 0.023cde	0.162 ± 0.011e	0.252 ± 0.007bcd	0.188 ± 0.056de	0.259 ± 0.048abcd	0.245 ± 0.016bcd	0.243 ± 0.009bcd	0.232 ± 0.008cde	0.276 ± 0.005abc	0.314 ± 0.042ab	0.332 ± 0.003a	0.249 ± 0.022bcd
Orn	0.926 ± 0.109d	0.888 ± 0.081d	1.216 ± 0.019d	8.456 ± 0.980b	8.790 ± 0.867b	3.920 ± 0.625 cd	8.890 ± 1.369b	9.495 ± 1.084b	3.252 ± 0.141d	23.855 ± 4.166a	20.811 ± 0.655a	8.070 ± 1.001bc
**taste aas**	13.751 ± 1.097**e**	13.647 ± 0.237**e**	18.208 ± 0.276**e**	90.414 ± 6.115**d**	103.433 ± 9.403**cd**	111.780 ± 10.676**cd**	124.911 ± 7.814**c**	121.815 ± 9.324**c**	125.337 ± 4.185**c**	244.429 ± 15.090**a**	227.238 ± 18.794**ab**	212.504 ± 19.219**b**
**Eaas**^b^	3.819 ± 0.064**e**	3.787 ± 0.098**e**	5.286 ± 0.097**e**	43.756 ± 2.523**d**	51.535 ± 6.096**cd**	55.794 ± 5.555**cd**	68.021 ± 5.400**c**	66.975 ± 6.614**c**	65.572 ± 2.917**c**	115.792 ± 12.166**a**	108.356 ± 8.599**ab**	96.008 ± 9.006**b**
**total aas**	15.205 ± 1.206**e**	15.049 ± 0.293**e**	20.759 ± 0.333**e**	102.662 ± 7.323**d**	116.914 ± 11.245**cd**	122.064 ± 11.520**cd**	141.044 ± 8.568**c**	138.157 ± 10.641**c**	135.586 ± 4.263**c**	279.319 ± 17.398**a**	257.099 ± 19.309**ab**	230.230 ± 20.996**b**
% taste^c^	90.439 ± 0.427b	90.686 ± 0.440b	87.714 ± 0.079c	88.086 ± 0.386c	88.501 ± 0.522c	91.569 ± 0.310ab	88.556 ± 0.845c	88.174 ± 0.049c	92.437 ± 0.213a	87.511 ± 0.663c	88.353 ± 0.658c	92.306 ± 0.075a
% Eaas^c^	25.229 ± 2.112d	25.163 ± 0.552d	25.464 ± 0.118d	42.672 ± 1.682bc	44.016 ± 1.000bc	45.695 ± 0.343ab	48.192 ± 1.085a	48.425 ± 1.126a	48.351 ± 0.828a	41.390 ± 1.927c	42.144 ± 0.982c	41.694 ± 0.195c

a Numbers mean the reference between parentheses: (1)^13^; (2)^14^; (3).^15^

b The summatory of threonine (Thr), lysine (Lys), isoleucine (Ile), leucine (Leu), valine (Val), tryptophan (Trp), histidine (His), and methionine (Met) was expressed as the total essential amino acid content (Eaas).

c The percentage was obtained by the division of the total content of taste-related/essential amino acids among the total content of amino acids.

Regarding bitter amino acids, a significant increment in Tyr, Phe, and Trp content is perceived in all sequential hydrolyzates compared to single hydrolyzates (*p* < 0.05), while significantly more concentration of Ile, Leu, and total bitter amino acids is observed only in Protana Prime hydrolyzates (*p* < 0.05). As shown in Figure 3A, it could be noticed that probe ultrasound pretreatment enhances significantly the content of total bitter amino acids only in single hydrolyzates (*p* < 0.05).

There is a significant increase of taste-related, essential, and total free amino acids (*p* < 0.05) in sequential hydrolyzates compared to single ones, being at least two times higher than single hydrolyzates in the case of Protana Prime hydrolyzates (Table 2). Flavourzyme also produces sequential hydrolyzates with the highest percentage of essential amino acids. Considering the effect of ultrasound, a significant reduction in the content of taste, essential, and total amino acids in the Protana Prime hydrolyzates is only observed in DHPUP in comparison with DHP (*p* < 0.05) which is also observed in Figure 3B if the free amino acid content is evaluated within groups. Therefore, it seems that probe ultrasound could reduce the release of total free amino acids in absolute terms. However, if the amino acid content is analyzed in a relative way, it is perceived that pretreatment with probe ultrasound produces single and sequential hydrolyzates with a higher percentage of taste amino acids compared to bath ultrasound pretreatment and without pretreatment, which could be considered a positive effect in the production of taste-rich hydrolyzates.

TAVs are shown in Table 3. Evaluating the enzyme action, the amino acids that most influence taste in unhydrolyzed samples are Glu, Lys, Ala, and Gly, which are related to sweet, bittersweet, and umami tastes. Nevertheless, in the case of BUP, the impact of Met and His on the taste is added. Therefore, the sulfurous, bitter, and bittersweet tastes of the samples are increased, which may not be desirable. Regarding single hydrolyzates, there is a significant increase in the TAV of almost all amino acids compared to the unhydrolyzed samples. The most taste-influencing amino acids are Glu, Lys, Val, Ala, and Met, which could be related to a predominantly umami, sweet, bittersweet, and sulfurous taste. As for the Flavourzyme hydrolyzates, the taste of Glu, Lys, and Ala stands as in the single hydrolyzates, and His, Phe, and Met taste increases significantly (*p* < 0.05). In view, Met being the amino acid that has the greatest TAV in Flavourzyme hydrolyzates could make the overall taste of these hydrolyzates more bittersweet and sulfurous, having an altered taste profile compared to the single hydrolyzates. Regarding Protana Prime hydrolyzates, Glu, Lys, Val, and Ala taste highlights as in the single hydrolyzates. If only free amino acids are considered as the main sources of taste, Protana Prime would generate hydrolyzates with a taste profile like the single ones, but with a significantly higher intensity (*p* < 0.05). Thus, it could be considered that Protana Prime sequential hydrolysis contributes to an enhancement of taste by maintaining their profile. Although probe ultrasound pretreatment significantly increases the taste impact of Arg and Tau in all groups (*p* < 0.05), this has less impact on the overall taste than the enzyme effect.

**Table 3:** Taste Activity Value of Liver Hydrolyzates (N=3, Mean±SEM).

		B	BUB	BUP	SH	SHUB	SHUP	DHF	DHFUB	DHFUP	DHP	DHPUB	DHPUP
amino acid	taste threshold (mg/100 mL)	mean ± SEM	mean ± SEM	mean ± SEM	mean ± SEM	mean ± SEM	mean ± SEM	mean ± SEM	mean ± SEM	mean ± SEM	mean ± SEM	mean ± SEM	mean ± SEM
Asp	100^a^^(2,3)^	0.913 ± 0.169c	0.902 ± 0.032c	0.924 ± 0.056c	**2.885 ±** 0.269bc	**3.449 ±** 0.424b	**2.838 ±** 0.311bc	**3.703 ±** 0.456b	**3.617 ±** 0.491b	**3.460 ±** 0.044b	**12.449 ±** 2.082a	**12.089 ±** 0.856a	**11.881 ±** 1.079a
Glu	30^(1,2,3)^	**6.547 ±** 0.646d	**6.475 ±** 0.069d	**7.118 ±** 0.158d	**20.857 ±** 2.059c	**26.839 ±** 3.187c	**28.889 ±** 3.132c	**28.582 ±** 1.658c	**27.375 ±** 2.313c	**30.225 ±** 0.964c	**62.852 ±** 8.310a	**53.060 ±** 0.598b	**56.225 ±** 5.281ab
Ser	150^(1,3)^	0.596 ± 0.090d	0.569 ± 0.027d	0.837 ± 0.014d	**3.759 ±** 0.408c	**4.142 ±** 0.499c	**3.974 ±** 0.213c	**4.570 ±** 0.293c	**4.520 ±** 0.243c	**4.501 ±** 0.199c	**8.949 ±** 1.127a	**7.456 ±** 0.391b	**8.710 ±** 0.818ab
Gly	130^(1,2,3)^	**1.841 ±** 0.280bc	**1.650 ±** 0.180c	**1.727 ±** 0.041c	**4.128 ±** 0.410bc	**4.423 ±** 0.418b	**2.758 ±** 0.122bc	**3.393 ±** 0.306bc	**3.311 ±** 0.255bc	**3.246 ±** 0.138bc	**13.991 ±** 2.747a	**12.487 ±** 0.257a	**14.012 ±** 1.022a
Thr	260^(1,2,3)^	0.157 ± 0.006e	0.166 ± 0.011e	0.307 ± 0.009e	**1.871 ±** 0.156d	**2.200 ±** 0.257d	**2.253 ±** 0.215d	**2.494 ±** 0.286d	**2.577 ±** 0.270d	**2.452 ±** 0.095d	**5.621 ±** 1.180b	**7.261 ±** 0.570a	**4.034 ±** 0.268c
Ala	60^(1,2,3)^	**2.397 ±** 0.411c	**2.414 ±** 0.288c	**3.078 ±** 0.019c	**15.980 ±** 2.157b	**16.965 ±** 2.176b	**15.210 ±** 1.528b	**16.633 ±** 0.846b	**16.683 ±** 1.010b	**15.250 ±** 0.497b	**44.484 ±** 7.853a	**42.851 ±** 9.245a	**34.381 ±** 3.032a
Arg	50^(1,2,3)^	0.083 ± 0.017f	**1.100 ±** 0.106def	**1.616 ±** 0.116d	0.784 ± 0.131def	0.470 ± 0.029ef	**4.120 ±** 0.801c	**1.678 ±** 0.092d	**1.298 ±** 0.131de	**10.830 ±** 0.478b	0.098 ± 0.004f	0.479 ± 0.052ef	**12.341 ±** 0.776a
Pro	300^(1,2,3)^	0.227 ± 0.027d	0.221 ± 0.014d	0.322 ± 0.011 cd	0.238 ± 0.013d	0.259 ± 0.020 cd	0.364 ± 0.010 cd	0.437 ± 0.004 cd	0.430 ± 0.013 cd	0.366 ± 0.011 cd	**1.233 ±** 0.221a	0.912 ± 0.131b	0.482 ± 0.037c
Val	40^(1,2,3)^	**1.659 ±** 0.010d	**1.603 ±** 0.109d	**1.414 ±** 0.150d	**21.160 ±** 0.859c	**21.200 ±** 2.875c	**23.804 ±** 2.422c	**18.396 ±** 1.425c	**16.969 ±** 1.210c	**16.882 ±** 1.632c	**53.117 ±** 6.208a	**52.073 ±** 8.959ab	**42.115 ±** 4.842b
Lys	50^(1,2,3)^	**2.769 ±** 0.156c	**2.655 ±** 0.086c	**3.238 ±** 0.040c	**21.506 ±** 2.353b	**24.553 ±** 2.317b	**19.432 ±** 1.948b	**27.140 ±** 2.621b	**26.343 ±** 3.067b	**24.858 ±** 1.603b	**67.863 ±** 14.308a	**58.925 ± 1**.037a	**61.468 ±** 6.218a
Tau	1877^(1)^	0.188 ± 0.010ef	0.117 ± 0.022fg	0.550 ± 0.051a	0.077 ± 0.000g	0.084 ± 0.010g	0.570 ± 0.053a	0.353 ± 0.008c	0.343 ± 0.009c	0.536 ± 0.022a	0.284 ± 0.007 cd	0.210 ± 0.045de	0.450 ± 0.022b
His	20^(1,3)^	0.320 ± 0.049d	0.302 ± 0.046d	**3.720 ±** 0.045c	**7.447 ±** 0.269b	**8.725 ±** 0.705b	**17.678 ±** 2.355a	**16.849 ±** 0.308a	**16.768 ±** 0.891a	**17.912 ±** 0.582a	**18.193 ±** 1.240a	**15.563 ±** 1.199a	**17.456 ±** 1.334a
Tyr	96.6^(1)^	0.265 ± 0.037e	0.242 ± 0.017e	0.420 ± 0.085e	**3.843 ±** 0.415d	**4.502 ±** 0.968 cd	**5.997 ±** 0.680bc	**7.617 ±** 0.590ab	**7.027 ±** 0.501ab	**6.991 ±** 0.676ab	**8.531 ±** 0.872a	**8.420 ±** 1.665a	**7.096 ±** 0.437ab
Met	30^(1,2)^	0.900 ± 0.117d	0.874 ± 0.062d	**1.401 ±** 0.041d	**9.964 ±** 1.084c	**11.492 ± 2**.406bc	**12.046 ±** 1.061bc	**36.146 ±** 2.893a	**36.022 ±** 4.269a	**35.195 ±** 1.182a	**17.695 ±** 3.131b	**14.615 ±** 2.759bc	**15.362 ±** 1.431bc
Ile	90^(1,2,3)^	0.468 ± 0.018e	0.490 ± 0.016e	0.439 ± 0.018e	**6.330 ±** 0.699 cd	**7.056 ±** 1.231 cd	**7.577 ±** 0.734c	**5.111 ±** 0.409 cd	**4.938 ±** 0.465 cd	**4.381 ±** 0.235d	**16.401 ±** 1.755a	**14.554 ±** 2.434ab	**13.000 ±** 1.408b
Leu	190^(1,2,3)^	0.445 ± 0.012d	0.448 ± 0.033d	0.452 ± 0.026d	**5.813 ±** 0.636bc	**7.428 ±** 1.504b	**8.173 ±** 0.791b	**4.273 ±** 0.342c	**4.156 ±** 0.384c	**4.134 ±** 0.188c	**14.883 ±** 1.678a	**13.440 ±** 2.385a	**11.863 ±** 1.089a
Phe	90^(1,2,3)^	0.395 ± 0.028e	0.413 ± 0.042e	0.352 ± 0.028e	**5.903**0.711d	**6.632 ±** 1.165d	**8.829 ±** 0.874 cd	**20.004 ±** 1.618a	**19.412 ±** 1.900a	**19.780 ±** 1.385a	**13.109 ±** 1.216b	**11.896 ±** 1.326bc	**10.542 ±** 1.029bc
Trp	90^(1)^	0.077 ± 0.017c	0.046 ± 0.007c	0.148 ± 0.012c	**1.784 ±** 0.390b	**2.127 ±** 0.155b	**3.086 ±** 0.318a	**3.366 ±** 0.467a	**3.478 ±** 0.202a	**3.364 ±** 0.207a	**3.848 ±** 0.552a	**3.529 ±** 0.230a	**3.288 ±** 0.277a

a Numbers between parentheses are the references in which the taste thresholds appears: (1)^13^; (2)^15^; (3).^16^

Protein hydrolyzates, especially those from animal sources, constitute interesting sources of bioactive peptides with biological potential. However, one of the major drawbacks when used in food is the appearance of bitter peptides, which may have a very negative effect on taste and therefore on consumer acceptance.^29^ These peptides usually have hydrophobic ends and a medium to small molecular size.^29^ Among the techniques for debittering hydrolyzates, physicochemical and biological techniques stand out. Physicochemical techniques such as ultrasound treatments and extraction with solvents or masking agents are generally not recommended because, although they are effective, their use can reduce the presence of peptides and amino acids, which results in a decrease of bioactive power and organoleptic properties of the hydrolyzate.^30^ Nevertheless, biological techniques such as sequential hydrolysis may give better sensory results and can also improve the bioactive potential.^31^ In this sense, sequential hydrolysis with endopeptidases such as Alcalase combined with exopeptidases like Protana Prime may favor the removal of hydrophobic ends and increase the degree of hydrolysis resulting in reduced size peptides that cannot fit into the bitter taste receptors.^29^ In fact, several studies confirm that this kind of combination exerts a good debittering effect.^32,33^ Flavourzyme is a combination of exo- and endopeptidases, which is why its effect may be lower than that of an endopeptidase enzyme such as Alcalase in conjunction with another exopeptidase such as Protana Prime. However, it is reported that Flavourzyme produces less bitter hydrolyzates than other enzymes such as Neutrase or Alcalase and that it can favor the appearance of a meaty taste;^34^ therefore, its use is also important to be taken into account despite its lower effect. Sweet, bittersweet, and umami-free amino acids can be considered to have a pleasant taste and their presence in food products may have a decisive influence on increasing the liking rating of consumers.^35^ Moreover, it has been shown that some umami amino acids, such as Glu or Asp, can exert a taste-enhancing effect in synergy with other compounds found in meat products, such as nucleotides.^13^ In addition, it is reported that umami contributes to the masking of bitter taste;^36^ therefore, its enhancement could have a very positive correlation with the increase in palatability of the hydrolyzates. Several publications have shown that ultrasound pretreatment improves the umami taste release of protein hydrolyzates.^28,34,37^ These authors attributed this to the disruption of the protein structure, which generates an increase in cutoff sites for the enzyme; however, if the pretreatment is intense, it has been observed that the proteins may tend to aggregate, producing the opposite effect, which would reduce the umami taste.^28^ This is consistent with the results obtained since, as shown in Figure 3, the umami amino acid content increased in the single hydrolyzates, while it did not increase in the sequential hydrolyzates, given that the longer hydrolysis time together with the ultrasound time may have favored protein aggregation. The increase in the degree of hydrolysis also occurs with sequential hydrolysis, and it is, therefore, to be expected that there is also an increase in umami taste in these, not only because of a higher release of Glu and Asp but also because it has been reported that hydrophilic peptides with very low molecular mass tend to have a positive correlation with this taste.^38^ Recently, several studies have reported that the combination of biological and physicochemical techniques, such as ultrasound with enzymatic hydrolysis, favors the debittering of hydrolyzates.^34,37^ However, no studies have been found that combine ultrasound pretreatment with sequential hydrolysis, and this strategy can be very appropriate, given that the pretreatment can improve the action of the endo- and exopeptidases. Nonetheless, it is necessary to know very well the parameters that influence the obtention of hydrolyzates rich in taste, since in this case, the power of the enzymes has overshadowed that of the ultrasound.

### 3.4 Biological Activity Assays

In the ABTS test (Figure 1B), it is observed that the most antioxidant samples are Protana Prime hydrolyzates, followed by the Flavourzyme hydrolyzates, single hydrolyzates, and finally the unhydrolyzed samples. On the other hand, analyzing the effect of ultrasound pretreatment, no significant differences are perceived within the groups (*p* < 0.05). The same trend is perceived in the DPPH (Figure 1C) and the ORAC (Figure 1D).

Observing the FRAP assay (Figure 1E), Protana Prime is again the enzyme that produces the most antioxidant hydrolyzates, and there are no significant differences between single and Flavourzyme hydrolyzates. The most noteworthy aspect of this assay is that the samples of sequential hydrolyzates pretreated with an ultrasound probe have significantly higher antioxidant activity (*p* < 0.05) than those pretreated with an ultrasound bath and those that were not pretreated.

Analyzing the ferrous chelating activity power assay (Figure 1F), hydrolysis reduces in general the chelating activity of the samples. Nonetheless, Flavourzyme hydrolyzates showed significantly higher values than single hydrolyzates (*p* < 0.05). In this assay, a significant increase (*p* < 0.05) in ferrous chelating activity is also observed in DHFUP and DHPUP. Therefore, it is considered that probe ultrasound does have a significant positive effect on this activity.

It is reported in several studies that the antioxidant activity increases in sequential hydrolysis of meat coproducts.^24,25^ These might be related to the increase in DH and therefore the release of small-size peptides (<1 kDa) that demonstrate its relationship with this activity.^39^ Moreover, the release of several antioxidant-free amino acids, such as arginine, methionine, or tryptophan,^40^ could also affect positively the antioxidant activity of hydrolyzates. Regarding ultrasound pretreatment, although several publications about its beneficial effect in the improvement of antioxidant activity in meat coproducts exist,^41,42^ in this study, its effect is, in general, lower than that of the enzymes which is why it can be overshadowed by them.

Iron is an essential micronutrient for human health, and its deficiency can lead to pathologies such as anemia. This deficiency can occur due to the low bioavailability of iron in its free form as a ferrous ion, which is why its chelation through its inclusion in peptides can favor its absorption in the organism.^43^ Furthermore, this free ferrous ion may have a pro-oxidant effect by promoting the generation of ROS, so its chelation may also contribute to improving the antioxidant status.^44^

It has been shown that the generation of peptides with chelating activity depends on the type of enzyme used, the hydrolysis conditions, and the raw material.^44^ Although this activity generally increases with the degree of hydrolysis, this is not always the case, since sometimes the protein of origin might already have this effect.^45^

Some studies indicate that ultrasound has a beneficial effect on ferrous ion chelating activity,^39,46^ although this is often associated with increased protease cleavage sites. However, in view of the results, this should not be the case, although it is possible that changes in the protein structure allow the release of different peptides that exert this chelating activity. In addition, the release of some free amino acids such as Tau^47^ or Arg^48^ could also be related to an increase in chelation. On the other hand, the type of ultrasound pretreatment that is applied has a relevant effect. Therefore, the ultrasound probe does exert a different effect from the ultrasound bath, generating sequential hydrolyzates with a high ferrous ion chelating capacity and with significantly higher antioxidant activity in the case of FRAP. This may be due to the lack of direct contact with the ultrasound emission source in the bath treatment, both the water and the container where the sample is immersed act as a barrier, reducing its effect while the contact with the sample is direct with the probe ultrasound, avoiding the barrier effect and increasing the action of the ultrasound.^49^

In summary, it could be stated that the choice of enzyme is probably the most relevant parameter to consider when generating hydrolyzates with good biological activities. However, the choice of ultrasound pretreatment does not seem to improve the effect of antioxidant activities, but it does favor the appearance of new ones; therefore, it is also important to take them into account.

In conclusion, the sequential hydrolysis of pork liver, especially the combination of Alcalase with Protana Prime in conjunction with a probe ultrasound pretreatment, generates hydrolyzates with a high taste capacity and a great capacity to exert biological activities. These hydrolyzates could be used in the future as a functional ingredient in the production of meat products, in which they could contribute to having a healthier profile and with fewer additives, since their inclusion would enhance the taste, thus being able to reduce the content of salts and sugars, and furthermore, due to its antioxidant activity, it is possible that it also exerts a preservative function.
